# A competitive edge: how social cues and spatial congruence influence joint attention in addressed or witnessed interactions

**DOI:** 10.1098/rsos.250467

**Published:** 2025-10-22

**Authors:** Isabel Blackie, Anisah Islam, Andrew Surtees, Mahmoud Medhat Elsherif

**Affiliations:** ^1^ University of Birmingham, Birmingham, UK; ^2^ Birmingham Women’s and Children’s Hospitals NHS Foundation Trust, Birmingham, UK; ^3^ University of Leicester, Leicester, UK

**Keywords:** addressed interaction, competition, cooperation, joint attention, witnessed interaction

## Abstract

Joint attention is crucial for the development of social cognition, but whether the type of relationship (i.e. cooperative or competitive) or interaction (e.g. addressed or witnessed) modulates joint attention is unclear. This study investigated these factors in 96 neurotypical adults using a video object-choice task. Here, participants chose between cups based on an actor’s pointing cue, either while being addressed or witnessing an interaction between two actors. Participants were primed about the actor’s cooperative or competitive intent. Experiment 1 found no significant interaction between these factors. However, Experiment 2, considering spatial attention (cue direction), revealed nuanced effects. In addressed interactions, more social cues led to faster responses, especially during spatially congruent cooperative trials. In witnessed interactions, responses were faster to spatially incongruent cues in cooperative trials and vice versa in competitive trials. These findings suggest that joint attention is not solely a passive response to social cues, but is actively shaped by the social context and spatial configuration, highlighting how individuals actively interpret and adapt to others’ attentional cues.

## 1. Introduction

Social interaction is underpinned by sophisticated cognitive abilities such as self–other differentiation, empathy and the ability to attribute mental states to others, which enable individuals to coordinate their actions and intentions with others [1,2]. One of the most important social skills defining these socio-cognitive abilities is joint attention (JA), as it provides a foundation for language acquisition, school readiness, educational attainment and the ability to sustain relationships [3–6].

Despite the developmental and evolutionary importance of JA, there is surprisingly a lack of consensus on what constitutes it and how to operationalize it within the literature. While overt JA behaviours (e.g. pointing) are necessary but not sufficient on their own to engage social interaction, social motivation and the opportunity to use these behaviours are also crucial [1]. For the current paper, JA is defined as the ability of two individuals to coordinate their attention on a shared object or event of interest using social cues (e.g. pointing; [7]). These social cues can simulate JA, whether on screen or in person. A key social factor defining JA is the context of communicative cues, which signal communicative intent from a communicator to an addressee or an audience [8]. For example, communicative cues (i.e. gaze alternating between partner and object or object only, while pointing) significantly benefit an infant’s ability to locate an object, whereas non-communicative cues (absent-minded gaze while staring at a watch) make learning an object’s location challenging [9,10]. This finding highlights the fundamental role of communicative intent in establishing JA. However, the definition of JA may be confounded by challenges with other socio-cognitive processes, such as processing facial expressions and eye contact (e.g. [11,12]), which can impact working memory [13]. While these processes are undoubtedly important for interactions where an individual is addressed directly, they may have less impact on types of interaction that do not depend on direct face-to-face contact (e.g. witnessing an interaction). Therefore, to disentangle JA from these potentially confounding socio-cognitive processes, it is crucial to examine JA across various types of interactions, not solely those involving direct address.

Universally, members of every society engage in two major types of interactions: either through directly interacting with others, or through witnessing/overhearing others interact. Till now, almost all of our knowledge about human joint attention across interaction types has come from work with infants and young children. In the Global North, children are more likely to be directly addressed, meaning one person communicates directly with them [14], while children in the Global South are more likely to learn in an environment where they share attention with others, without addressed interaction (i.e. overhearing; [15–17]). However, this does not mean that children from either culture do not encounter the other type of interaction (e.g. [18–21]). Notably, laboratory-based research has shown that 18-month-old infants from the Global North can learn new words via both addressed interactions and non-addressed (witnessed) interactions, such as overhearing (e.g. [14,22,23]; see review by Akhtar *et al*. [24]), and gestural following (pointing: [9,10]; gaze following: [25]). These studies suggest that children primarily acquire linguistic and social knowledge through one of these communicative interactions [18]. However, overhearing may be less influential than addressed interaction for vocabulary size (see [26,27]).

Across varied social-cognitive processes, it has more recently been acknowledged that understanding continuity and discontinuity between children and adults is crucial. It helps us establish a putative endpoint for children’s development, comprehend why some children experience difficulties and map individual differences in adulthood (e.g. in mindreading; see review by Apperly [28]). This logic also applies to joint attention. Currently, the comparison between addressed and witnessed interaction is predominantly limited to child samples. The existing adult literature on this topic appears to present a nuanced picture. Fox Tree [29] observed that adults are more accurate in following instructions as overhearers than addressed interaction. However, it is important to note that the majority of addressed interactions in real-world settings involve additional social cues and processes (e.g. direct eye gaze, facial expressions and shared contexts), which are absent in witnessed interactions [24]. Conversely, adults benefit from addressed interactions during tasks that involve perspective taking and recognizing emotions and facial expressions (see review by Frith & Frith [30]). Therefore, further research with adults is necessary to contextualize the differences between addressed and witnessed interactions in JA. Given that adults appear to benefit from overhearing dialogues more than monologues, and that overhearing is reflective of real-life learning, the current work aims to investigate whether witnessing non-verbal communicative interactions modulates JA in adults’ ability to learn about the social world. We hypothesize this could allow them to process information more efficiently while other cognitive demands are reduced (e.g. working memory, eye-gaze processing and the interpretation of facial expressions; [11–13, 31]).

Alongside the variety in external structure of JA (directly addressed versus overhear), JA varies with respect to the interactional context in which it occurs. While JA appears inherently cooperative, it can also be influenced by competitive dynamics. To examine this, Herrmann & Tomasello [32] used the object choice task (OCT), an explicit decision-making task where the cued individual must locate an item in one of two cups, based on the actor’s non-verbal cue (e.g. pointing or gaze). The cuer either pointed to the correct container (i.e. informing condition) or held out their arm towards the correct container and told the participant firmly ‘Don’t take this one’; prohibiting condition. The results indicated that 24-month-old infants performed better in the informing condition than the prohibiting condition, while 18-month-old infants performed at chance. The authors concluded that infants need to understand a cooperative communicative motive. Although the adults’ instruction to prevent access to a desired resource validly represents a goal-hindering or competitive interaction within that experimental context, creating a genuine competition for resources [33]. Competition, in Herrmann & Tomasello’s [32] context, arises from a direct prohibition. By contrast, many human–human competitive interactions, involve divergent goals within a shared task where individuals actively work against each other’s success to achieve a relative win, as opposed to a direct prohibitive stance [34]. Therefore while Herrmann and Tomasello’s manipulation effectively establishes a competitive motive, its specific dynamics differ from scenarios where agents actively hinder another’s success within a shared task structure. Our study extends this foundational understanding to adult participants, examining how these fundamental social interaction types (cooperation versus competition) influence joint attention, even when direct inferences about intentions are readily made.

The role of competition and cooperation has been largely neglected in the study of JA in adults. To our knowledge, only one recent study has extended the cooperative and competitive paradigm to adults [35]. Ciardo and colleagues exposed participants to a task where actors displayed either cooperative or competitive behaviours. The authors found that highly competitive individuals were cued equally by both cooperative and competitive faces, whereas those low in competitiveness were cued more by competitive faces. The authors argued that highly competitive individuals may be more attuned to social cues, regardless of the context, as they are constantly seeking information to gain an advantage. In cooperative contexts, this heightened sensitivity may lead them to monitor both cooperative and competitive actors, as the actions of competitors could potentially disrupt their goals. However, in competitive contexts, the distinction between cooperative and competitive actors becomes less relevant, as all social cues are potentially valuable for gaining an edge. This means that JA behaviour should be more pronounced in competitive contexts relative to cooperative contexts. To investigate the possibility that JA behaviours in adults are strengthened by competitive scenarios, we used the OCT to investigate JA in both a competitive and cooperative scenario. Here we manipulated the level of social communicativeness of cues, while controlling for hand configuration. We also manipulated the spatial congruence of cues to test whether spatial, rather than social, processes underpinned the observed JA behaviour. To encourage a more competitive scenario, the cuer either competed directly with the participant or the other actor by pointing to the incorrect cup.

This study explored how social cues and spatial attention influence decision-making in cooperative and competitive contexts. We used a within-participant design with two distinct versions of OCT: an addressed OCT (a-OCT), where participants directly interacted with an on-screen agent, and a witnessed OCT (w-OCT), where participants observed interactions between two agents. Both versions were designed to be visually comparable, and we used age-matched actors of diverse genders and ethnicities to enhance generalizability [36]. Our experimental manipulations focused on three key factors: cue type (high social versus low social), task (addressed versus witnessed), and intention (cooperative versus competitive). In the high-social cue condition, an agent used direct pointing and alternating gaze to intentionally communicate the ball’s location. Conversely, the low social cue condition featured incidental pointing, where the agent appeared preoccupied with a phone, providing a similar spatial cue but lacking direct communicative intent. Participants were informed that cooperative agents would indicate the correct location, while competitive agents would intentionally misdirect them. Our primary measure was response time (RT). The specific predictions regarding the effects of these manipulations on participant behaviour are outlined below.

The present investigation is the first, to our knowledge, to address whether competition and cooperation moderate joint attention in an addressed and witnessed non-verbal environment, using an OCT (see reviews [37,38]). We expect faster responses to more communicative cues (labelled ‘high social’) compared with less communicative cues (labelled ‘low social’) [10]. Similarly, we anticipate faster responses to addressed interactions compared with witnessed interactions [39]. Furthermore, we hypothesize an interaction between communicative cue, modality and intention. Here, we predict that in the addressed condition, where individuals can directly respond to the social cues, high-social cues will have a greater impact on competitive behaviour compared with cooperative cues [35]. However, in the witnessed condition, where individuals cannot directly interact with the cued individual but witness the interaction between the actors, the difference between high-social and cooperative-competitive cues may be less pronounced.

## 2. Experiment 1

### 2.1. Methods

This study was pre-registered on the Open Science Framework (https://osf.io/2tkm7).

#### 2.1.1. Transparency and openness

We report how we determined our sample size and all data exclusions, manipulations and measures below. Data for Experiment 1 were collected in 2022−2023, and data for Experiment 2 were collected in 2023−2024.

#### 2.1.2. Participants

A power analysis was conducted using G*Power software (version 3.9.1, [40]), which determined 45 participants were required to see a significant three-way interaction between our variables (see electronic supplementary material, appendix A). To ensure this was possible, 60 undergraduates were recruited and reimbursed with course credits. The experiment was approved by the University of Birmingham’s Science Technology Engineering and Maths Ethics Board and conducted in line with the British Psychological Society’s ethical guidelines (ERN_09-719F). Participants had to be at least 18 years of age, have normal or corrected to normal vision, and fluent English. Fifteen participants were excluded due to video lagging (*n* = 2) or performing below 80% accuracy (*n* = 13). Our final sample consisted of 45 participants aged between 18 and 39 (*M* = 23.22) of varying socio-economic statuses; for full demographic information see table 1.

**Table 1.** Demographic characteristics of our final sample in Experiment 1.

demographic variable		*n*	%
gender	male	16	35.56
	female	24	53.33
	non-binary	3	6.67
	transgender	2	4.44
handedness	right	36	80
	left	5	11.11
	ambidextrous	4	8.89
bilingual	yes	25	55.56
	no	20	44.44
glue ear	yes	0	0
	no	45	100

#### 2.1.3. Design

This study used a within-participant design comprised of three factors, each with two levels: cue type (high social, low social), task (addressed, witnessed) and intention (cooperative, competitive). The primary outcome measure was Response Time (RT), measured in milliseconds from decision screen onset until response.

##### 2.1.3.1. Object choice task

Two versions of the task were administered, an a-OCT (modelled from Behne *et al*. [9]), and a w-OCT [10]. Both were comparable on visual complexity to ensure low level visual properties did not drive any pattern. Additionally, actors were age-matched, and of differing genders and ethnicities to reduce any intrinsic biases and increase generalizability [36]. A Latin square design was employed to avoid actor repetition but ensure data collection for each actor across all conditions. Each OCT was split into four practice trials followed by 24 experimental trials (four blocks of six trials). R statistical software [41] was used to confirm internal consistency of each OCT, a-OCT (*α* = 0.91) and w-OCT (*α* = 0.93).

##### Addressed OCT

**Figure 1.**
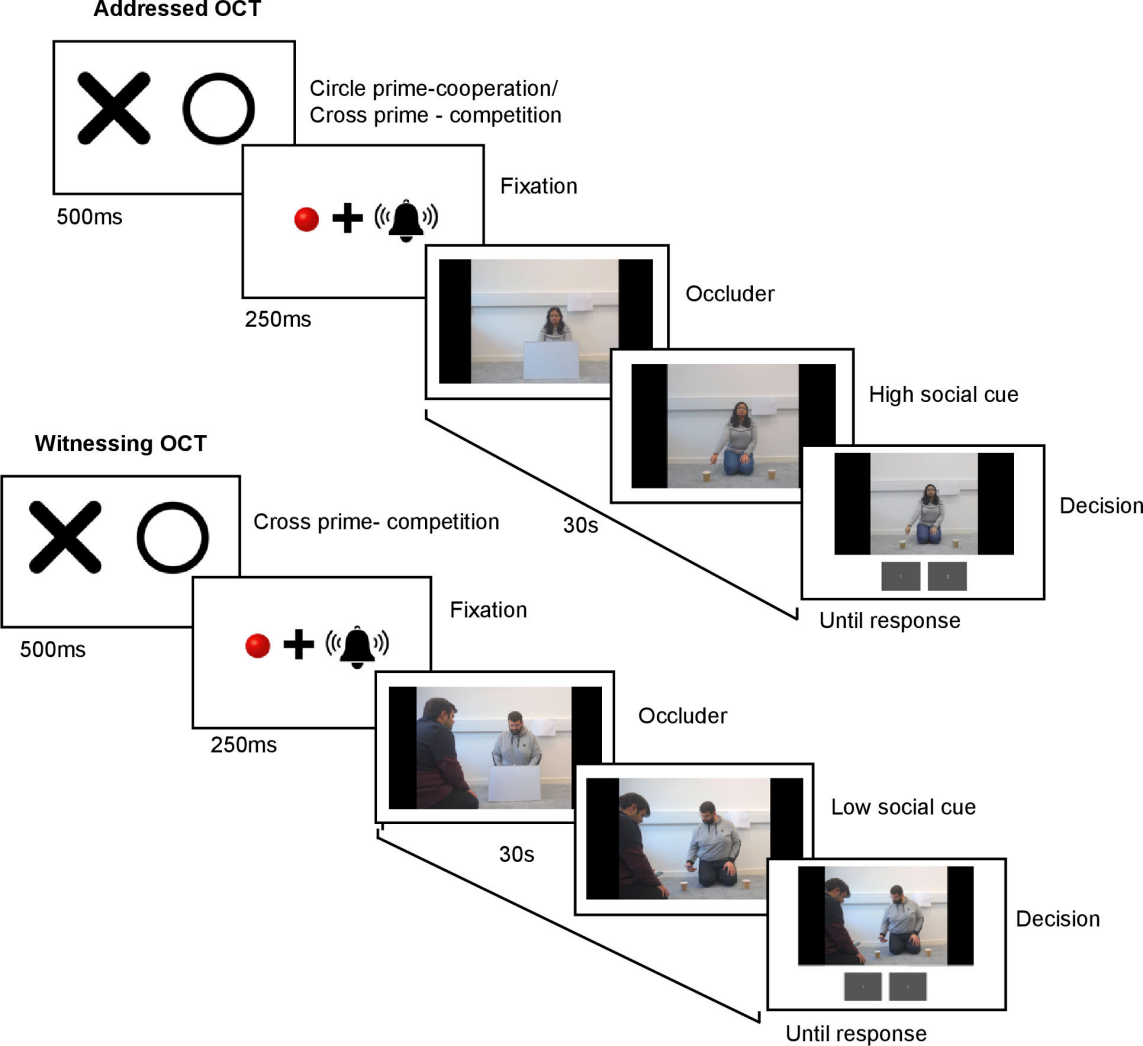
The experimental structure of the addressed and witnessed object choice task paradigm in Experiment 1. In the addressed object choice task (OCT) trial here, the correct decision would be to select the cup on the left-hand side of the screen as the actor is cooperating with participants. In the w-OCT trial, the correct decision would be to select to the cup on the right-hand side of the screen as actor is competing with the partner. Circle prime was reflective of cooperative cues, whereas cross primes were representative of competitive cues. The individual in this manuscript has given written informed consent to publish these case details. All actors have given written informed consent to publish the videos and pictures on the OSF.

Practice trials began with a central prime: a black circle reflective of cooperative intent where participants must select the item pointed towards or a black cross representative of competitive intent where participants must select the opposite cup that the cuer is pointing towards (see figure 1 for experimental structure for a-OCT). A red ball and attractive jingle were then displayed to engage participants’ attention, before a video appeared showing an actor kneeling centrally with two brown cups (7 × 8 × 9 cm) placed side-by-side in front of them. The distance between the actor and cups was varied to minimize the effects of spatial attention. The actor then showed the ball to the camera and hid it in one of the cups asking afterwards ‘Can you find the ball?’. The actor then indicated the ball’s location using a high or low social cue. Participants knew this would cue the correct location if actors were cooperators, but it would cue the incorrect location if actors were competitors. For high-social cues the actor pointed directly at the cup and alternated their gaze between the cup and the camera, an example of intentional communication [42]. For low social cues the actor held their phone out in the direction of the cup and appeared preoccupied with it, an example of incidental pointing, a gesture with similar spatial salience but without conventional communicative value [43]. Crucially, while not explicitly communicative, the ‘low social cue’ condition still provided social information derived from the mere presence and actions of another human agent within the scene, serving as a baseline for general social processing compared with the explicit communicative engagement of the ‘high-social cue’. We rigorously controlled for non-social confounds, ensuring high- and low-social cues were matched on dimensions such as the distance between the agent and the target object, with only the presence/absence of communicative social engagement varying. Next, a still image of the cue was shown with two boxes corresponding to each cup for participants’ decision. Participants then received feedback; if correct the actor said ‘Oh wow you found the ball!’, if incorrect they said ‘Oh nice try, it was in here’, revealed the ball’s correct location and said ‘Let’s try again’.

Experimental trials had a similar format; however, once the video started actors showed the two cups to the camera and placed them behind an opaque occluder (60 × 40 cm), which they remained behind until the ball was hidden. Once hidden the occluder was removed, and a high- or low-social cue was used to indicate the ball’s location. These trials were completely non-verbal, so participants were not prompted to locate the ball and no feedback was given.

##### Witnessed OCT

The w-OCT practice trials followed a similar format to a-OCT practice trials; however, two actors were present in the video, the cuer and a partner (see figure 1 for experimental structure for w-OCT). The cuer was knelt in the same position as before and the partner knelt to the side, the side they knelt was alternated. Throughout the trial the cuer only communicated to the partner, they did not look at the camera. High- or low-social cues were directed towards the partner and once given partners turned to the camera and asked ‘Can you help me find the ball?’. Cues were similar to the a-OCT, but in low-social cues the partner also got their phone out holding it just in front of them. Decisions were made in the same way and feedback was given as per a-OCT practice trials.

The experimental structure for w-OCT trials were similar to the a-OCT experimental trials but the two actors interacted with each other non-verbally and never looked at the participant directly.

##### 2.1.3.2. Revised competitive index questionnaire

Data were also collected from the revised competitive index questionnaire (r-CIQ) [44] for exploratory analyses. The r-CIQ is a 14-item Likert scale questionnaire to measure participants’ competitiveness, included for exploratory analyses as Ciardo *et al.* [35] found competitiveness modulated the effect of social relationships on JA. It included statements like ‘I like competition’, which participants rated on a five-point scale from strongly disagree to strongly agree.

##### 2.1.3.3. Raven’s standard matrices

The Raven’s standard matrices test assessed non-verbal intelligence [45]. Participants were shown 60 patterns of increasing complexity and had to select which pieces completed each complex pattern. Gorilla (Gorilla.sc) software was used to implement this task. The number of correct answers was recorded and no time limit was set.

##### 2.1.3.4. British picture vocabulary scale 3rd edition

The British picture vocabulary scale (BPVS) was used to measure the participants’ receptive vocabulary [46]. Participants heard a word and selected the corresponding picture from a choice of four. Each participant completed six vocabulary tests (sets 9−14 with 12 words per set). Gorilla (Gorilla.sc) software was used to implement this task.

##### 2.1.3.5. The comprehensive autism traits questionnaire

The comprehensive autism traits questionnaire (CATI) was used to measure whether joint attention relates to real-world experiences and social differences in the general population [47]. It is a self-report questionnaire with 42 items designed to measure traits and characteristics related to autism in the general population. These items were evaluated on a five-point Likert scale from strongly disagree to strongly agree. The scale is divided into the following subscales: repetitive behaviour (REP), cognitive rigidity (RIG), sensory sensitivity (SEN), social interactions (SOC), communication difficulties (COM) and social camouflaging.

### 2.1.4. Procedure

Participants completed two sessions via Zoom (https://zoom.us/) to complete each OCT: witnessed and addressed. Each individual session, including instructions, task completion and post-task assessments, lasted approximately 1 h. The administration of these tasks to the same participants was deliberately separated by 3–7 days (*M* = 5.93). This was done to minimize potential carryover effects, learning and fatigue that could arise from presenting two distinct social cognitive tasks, each requiring different cognitive perspectives, within a single experimental session. Therefore, the total duration for each participant across the entire study, considering the 3−7 day interval between sessions, was approximately 2 h.

To mitigate potential order effects, the sequence of the witnessing and addressed tasks was systematically counterbalanced across participants. Approximately half of the participants completed the witnessing task first, followed by the addressed task, while the remaining half completed the addressed task first, followed by the witnessing task. This explicit counterbalancing strategy ensured that any residual order effects were evenly distributed across conditions and did not systematically bias the results.

Upon joining each Zoom, call participants received standardized instructions for the relevant OCT by researchers. A secure link to the experiment, hosted on Gorilla (https://gorilla.sc), was then provided via the chat function. The decision to conduct this experiment online was carefully considered, driven by several factors. During the initial data collection period (2022−2023), the ongoing concerns regarding COVID-19 made online administration a priority for the health and safety of our student participants. For subsequent data collection in Experiment 2 (2023−2024), the online format was maintained to ensure consistency with the initial dataset and to facilitate access to a broader, more diverse participant pool than typically accessible in a single laboratory setting, thereby enhancing the generalizability of our findings.

In the participant’s first session, an information sheet, consent form and a demographic questionnaire, collecting data on questions about age, gender, race and handedness were completed, along with the CATI and the r-CIQ. These questionnaires were administered only once during the first session. Before the practice trial for each OCT, brief instructions were shown, which were reiterated before experimental trials. Breaks were provided after each block of the experimental trials, but participants could pause on the ready screen of each trial if needed. Participants were instructed to use the mouse, using their dominant hand, to select either 1 or 2 on the screen which corresponded to the left or right cup, respectively. Trials were presented in a pseudo-randomized order, which ensured there was an equal number of each cue type, social relationships, and left and right cues, and that each condition would not be presented more than twice in a row. After each OCT, they were given either BPVS or Raven’s progressive matrices. Upon completion, participants were debriefed and received university course credits once they had participated in both sessions.

### 2.1.5. Data analysis

Our analyses, outlier rejection, missing data and inference criteria were pre-registered https://osf.io/2tkm7. Data processing and all statistical analyses were conducted in R version 1.3.0 [41]. Data were analysed by means of general linear mixed models, using the lmer function in the lme4 package version 1.1.26 [48] and ‘tidyverse’ package [49] in R 3.6.1 [41]. All following statistical tests were two-tailed. The statistical tests were significant at *p* < 0.05 if the *t*-value is greater than 1.96 [50].

To compare whether there was a difference between cooperative and competitive interactions in addressed and witnessed conditions, a general linear mixed model (GLMM) was conducted. The model included the following within-participants factors: intention (cooperation and competition, sum-coded with cooperation as the intercept), task (addressed and witnessed, sum-coded with addressed as the intercept) and cue type (communicative, non-communicative, sum-coded with non-communicative as the intercept). The fixed effects were allowed to vary randomly by participant and item for all slopes and intercepts [51]. All continuous variables were centred prior to analysis. The RTs and accuracy were used as dependent variables.

We started with a maximal model, including random participants, item intercepts and random participant slope. However, maximal models do not always converge. If there was no convergence, we excluded random slopes for participants and, if necessary, simplified to non-random models until convergence. Here, we checked the assumptions of general linear mixed models following the recommendation by Meteyard and Davies [52]. We applied an inverse transformation (−1000/RT) to make the residuals of raw reaction times more normally distributed, and to ensure there was no heteroscedasticity and no deviations from linearity.

The minimal model in the fixed-effects structure was isolated using the drop1 function, which identifies the most complex fixed effects explaining the least variance. Fixed effects were removed until the model with the minimal Bayesian information criterion (BIC) was reached [53]. ΔBIC implies the difference between the full model and reduced model; a positive ΔBIC indicates that the reduced model is better than the null model. We have included Bayes factor (BF) approximations, using the formula (exp(ΔBIC/2) [54]; by using the BF, we compared the relative evidence for different models. For instance, a BF value of 5 implies that the reduced model is five times more likely than the full model. In general, the higher the ΔBIC and BF, the more likely the reduced model is in comparison with the full model. Based on these tests, we created a minimal model, which included the combination of fixed effects that provided the best fit of our data.

### 2.1.6. Exploratory analyses

We investigated the correlation between a measure of competition, as assessed by the revised competition index questionnaire, and a competition index derived from the subtraction of the RTs in addressed and w-OCTs to ensure the competitive cue was valid.

### 2.2. Results

Following the methods of Loenneker *et al.* [55], we pre-processed our data to ensure reliability and accuracy. First, we conducted a quality check on all participant data. We excluded trials with RTs under 200 ms, as these typically indicate impulsive or anticipatory responses. A high number of such trials could suggest a lack of task engagement. After this step, we calculated the proportion of correct responses for each participant. Participants who produced more than 20% errors were removed from the analyses in either a-OCT or w-OCT; however, 13 participants mets this exclusion criterion. All the data were included for accuracy analyses, while only correct trials were included in the RT analyses. In addition, we trimmed RTs, by removing any responses ± 2.5 s.d. or above from participant’s mean RT. This led to 6.81% of the data being removed in total. Average RTs, s.d.s and the proportion of correct responses for each condition are shown in table 2.

**Table 2.** Mean response times and proportion correct for task, cue type and intention.

	addressed		witnessed	
	cooperation	competition	cooperation	competition
high social				
RT	1997 (959)	2116 (826)	2085 (1077)	2512 (1113)
accuracy	0.96 (0.21)	0.95 (0.21)	0.97(0.18)	0.93 (0.26)
low social				
RT	2148 (945)	2246 (925)	2319 (1063)	2297 (1038)
accuracy	0.97 (0.17)	0.93 (0.25)	0.99 (0.12)	0.94 (0.23)
social effect (RT)	151	130	234	215
social effect (accuracy)	0.01	−0.02	0.02	0.02

Note. RT: response times. Response times are measured in milliseconds and standard deviations are in parentheses. Positive priming effects reflect facilitation for targets preceded by high-social, relative to low-social, conditions, such that there are faster RTs and higher accuracy for high-social cues than for low-social cues. Conversely, negative values indicate faster RTs and higher accuracy for low-social cues than for high-social cues, which is indicative of inhibition.

#### 2.2.1. Accuracy

All the data were included for accuracy analyses. Performance was at ceiling, making it difficult to compare conditions. We will therefore not discuss accuracy data in any more detail.

#### 2.2.2. Reaction times

We first investigated whether there was a speed–accuracy trade-off. There was no significant correlation between RTs and accuracy (*r* = −0.22, *p* = 0.15) indicating there was no speed–accuracy trade-off, thus RTs could be used for analyses.

We observed that the model for the first experiment did not converge until the item slope was removed, leaving only an intercept and only the condition was included as an individual factor by itself in the random structure for the participant. The output of this model is shown in table 3.

**Table 3.** The minimal model output for RTs for Experiment 1.

fixed effects	estimate	std. error	95% CI	95% UCI	*t*-values
(intercept)	−0.5659	0.0262	−0.6193	−0.5117	−21.60^*^
*manipulation*					
intention	0.0587	0.0092	0.0015	0.0415	6.35^*^
task	0.0092	0.0201	−0.0101	0.02768	0.46
cue type	0.0599	0.0143	−0.0880	−0.0472	4.19^*^

Note. * *p* < 0.05. UCI: upper confidence interval.

##### 2.2.2.1. Confirmatory analyses

The reduced model was significantly different from the full model (full model BIC: 157.75; reduced model = BIC: −204.43, *p* < 0.001, ΔBIC: 362.17, approx. BF > 10 000), thus the final model is based on the reduced model. We did not observe the expected interaction between cue type, intention and task in the model. In the reduced model (table 3), we observed that there was a significant effect of cue type on reaction time latencies such that participants responded more quickly to high-social than low-social pointing. In addition, there was a significant effect of social intention on inverse reaction time such that participants responded more quickly to cooperative cues than competitive cues.

##### 2.2.2.2. Exploratory analyses

We also conducted exploratory correlational analyses between the r-CIQ scores and competitive effects from each OCT. Competitive effects were calculated by subtracting competitive mean reaction times from cooperative mean reaction times for each OCT. A significant moderate negative correlation was found between the addressed competitive effect and r-CIQ scores (*r* = −0.44, *p* = 0.003), meaning those scoring low in self-reported competitiveness were more likely to respond slower to competitive stimuli. There was no significant correlation between competitive effect and CATI (*r* = 0.25, *p* = 0.10), BPVS (*r* = 0.11, *p* = 0.49) nor Raven (*r* = 0.02, *p* = 0.92). However, there was no significant correlation between witnessed competitive effect and r-CIQ scores (*r* = 0.11, *p* = 0.47), CATI (*r* = −0.17, *p* = 0.26), BPVS (*r* = −0.01, *p* = 0.95) nor Raven (*r* = 0.03, *p* = 0.84).

### 2.3. Discussion

In Experiment 1, we investigated the processing of cooperative and competitive intentions within both addressed and witnessed conditions. We observed a significant cue type effect on RTs, with participants responding more quickly to high-social cues (i.e. pointing) than low-social cues (i.e. pointing with the mobile phone). This finding aligns with prior research on infant pointing behaviour [9,10], suggesting that the social effects of pointing is primarily driven by its communicative properties. Interestingly and contrary to our initial hypotheses, we observed that cooperative cues were responded to more quickly than competitive cues. This finding, observed across both addressed and witnessed interactions, aligns with the concept of shared representations during task performance. When individuals work towards a common goal, the alignment of intentions, as in cooperation, may facilitate faster processing of the social cue, even when not directly interacting with the participant (e.g. [30]).

Counterintuitively, we expected there to be stronger differences between addressed and witnessed interaction due to self-relevance and direct gaze (e.g. [13]), our results suggest that the salience of processing the cooperative and competitive cues itself may have overshadowed these distinctions in our specific task. This could be due to the intrinsic human ability to extract social information from observed interactions, regardless of direct involvement. Furthermore, while the high- and low-social cues might seem unsurprising given the arousal associated with direct gaze, it is crucial as it validates our manipulation and establishes a necessary baseline for direct communicative engagement.

Despite these findings, we did not observe the predicted three-way interaction between task, cue type and intention, as predicted. One possible explanation for this is that both gaze and pointing cues elicit reflexive attention shifts towards the cued location [56–58]. Given that cooperative cues were always spatially congruent with the correct location, while competitive cues were incongruent, this inherent congruence might have masked the expected difference, preventing competitive cues from being responded to more quickly than cooperative cues.

To further investigate the role of spatial congruence, Experiment 2 will systematically manipulate the spatial congruence of cooperative and competitive cues in both addressed and witnessed interactions. If spatial congruence is indeed the underlying factor, we predict no difference between cooperative and competitive cues in the spatially congruent condition for high-social pointing gestures. Conversely, we expect a larger difference between high- and low-social pointing gestures in the spatially incongruent condition. This is because pointing, in this context, becomes more effortful and is rooted in its developmental function as a cooperative gesture [59]. Regardless of spatial congruence, we predicted no difference between cooperative and competitive cues in witnessed conditions, as the actor is not interacting directly with the cuer.

## 3. Experiment 2

### 3.1. Methods

This experiment was also pre-registered on the Open Science Framework (https://osf.io/8dvpw).

#### 3.1.1. Participants

Like Experiment 1, a power analysis was conducted, which determined 48 participants would be needed to see a significant interaction between our variables. Seventy-three participants were recruited and reimbursed with course credits. None of the participants participated in the previous experiment. The experiment was approved by the University of Birmingham’s Science Technology Engineering and Maths Ethics Board and was in full compliance with the British Psychological Society’s ethical guidelines (ERN_09−719F). The same inclusion criteria were applied as Experiment 1. Twenty-five participants were excluded due to video lagging (*n* = 15) or performing below 80% accuracy (*n* = 10). Our final sample consisted of 48 participants aged between 18 and 38 (see table 4 for full demographic details). Participants in Experiment 2 did not significantly differ from participants in Experiment 1 in terms of individual differences (see table 5).

**Table 4.** Demographic characteristics of our final sample in Experiment 2.

demographic variable		*n*	%
gender	male	8	17
	female	39	81
	non-binary	1	2
handedness	right	41	85
	left	5	10
	ambidextrous	2	4
bilingual	yes	26	54
	no	22	46
glue ear	yes	0	0
	no	48	100
ethnicity	Arab	5	10
	Asian	6	13
	Black	6	13
	South Asian	9	18
	White	22	46

**Table 5.** Variable comparison between Experiments 1 and 2 samples*.* BPVS, British picture vocabulary scale; CATI, comprehensive autistic trait inventory. Standard deviations are in parentheses.

variables	Experiment 1	Experiment 2	*t*(d.f.)	*p*
competition	41.96 (13.49)	42.31 (10.06)	*t*(91) = 0.25	0.80
Raven	43.38 (10.15)	40.99 (9.93)	*t*(91) = 1.16	0.25
BPVS	46.64 (10.32)	46.23 (9.18)	*t*(91) = 0.26	0.80
CATI	117.88 (27.27)	122.83 (26.18)	*t*(91) = −0.86	0.39

#### 3.1.2. Design

Experiment 2 used the same within-participant design as Experiment 1, except with an additional factor of spatial attention congruence, as the previous manipulation of cooperation and competition was confounded with spatial attention. Consequently, the observed differences may have been the result of spatial, as opposed to social, processing. This factor had two levels, congruent or incongruent. The same outcomes as Experiment 1 were measured.

#### 3.1.3. Materials

**Figure 2.**
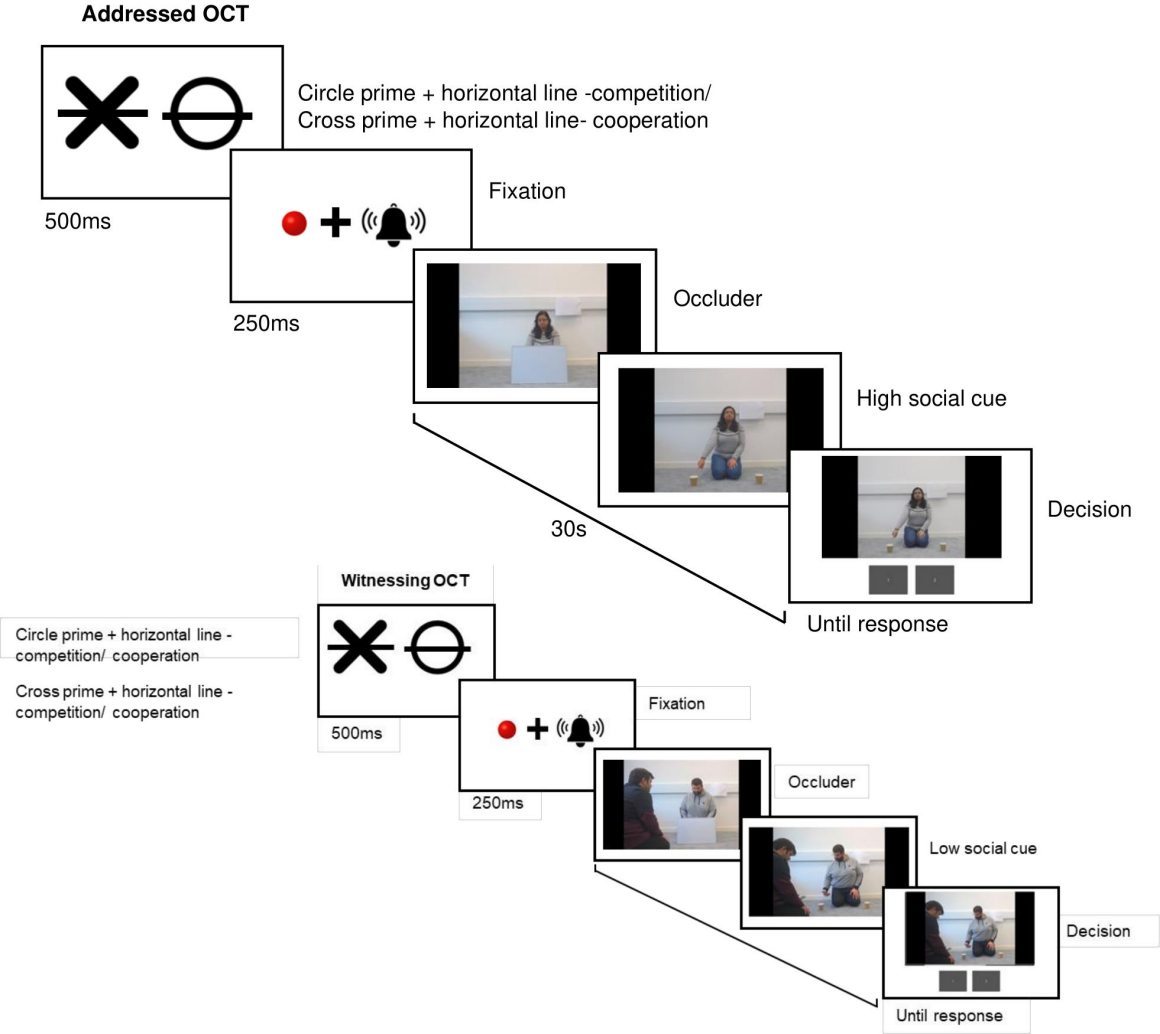
The experimental structure of the addressed and witnessed object choice task paradigm in Experiment 2. In the addressed object choice task (OCT) trial here, the correct decision would be to select the cup on the right-hand side of the screen, the actor is pointing to the empty cup on the left, so you must select the right cup. In the w-OCT trial, the correct decision would be to select the cup on the left-hand side of the screen as the actor is trying to trick you by pointing to the cup with the ball in it. The individual in this manuscript has given written informed consent to publish these case details. All actors have given written informed consent to publish the videos and pictures on the OSF.

The same OCT was administered in Experiment 2 except we modified the OCT to incorporate the factor of spatial attention congruence. Here we introduced two additional prime types: line or no-line. A circle with no horizontal line informs the participants that the actor is cooperating with them to locate the baited cup. A circle with a horizontal line through it indicated that the actor would cooperate with the participant by indicating the location of the empty cup, thus participants would need to remember this and select the other cup to be successful. The cross with no horizontal line informs participants not to trust the cuer and to choose the opposite cup, while the cross with the horizontal line tells the participant to follow the cuer as they are double bluffing the participant. Hereon, we define line or no-line as spatially congruent and incongruent. Spatially congruent primes are the no-line circle and line cross, spatially incongruent primes are the line circle and no-line cross. With these additional primes the number of trials increased, with eight practice trials being followed by 48, as opposed to 24, experimental trials in both the a-OCT and w-OCT. See figure 2 for an example trial in both the a-OCT and w-OCT with these new primes being used.

#### 3.1.4. Procedure and data analysis

The same procedure and data analytic techniques were used as before except that participants were shown a diagram of the new primes and their descriptions to aid understanding before the experimental trials.

### 3.2. Results

Following the same outlier exclusion criteria as Experiment 1, we removed 3.21% of the RT data in total. Average RTs, s.d.s and the proportion of correct responses for each condition are shown in table 6.

**Table 6.** Mean response times and proportion correct for task, cue type and intention.

	addressed				witnessed			
	cooperative		competitive		cooperative		competitive	
	con	incon	con	incon	con	incon	con	incon
high social								
RT	1962 (2211)	2469 (2808)	2226 (1767)	1968 (1452)	1870 (1392)	2025 (1497)	2352 (1887)	2019 (1901)
accuracy	0.95 (0.22)	0.94 (0.24)	0.93 (0.25)	0.94 (0.22)	0.96 (0.19)	0.92 (0.27)	0.98 (0.15)	0.92 (0.28)
low social								
RT	2219 (1933)	2434 (1900)	2534 (2339)	2456 (3474)	2192 (1972)	2580 (2123)	2615 (2574)	2210 (1818)
accuracy	0.97 (0.18)	0.92 (0.27)	0.93 (0.25)	0.93 (0.24)	0.92 (0.27)	0.95 (0.22)	0.92 (0.28)	0.94 (0.24)
social effect (RT)	257	−35	308	488	322	555	263	191
social effect (accuracy)	0.02	−0.02	0.00	−0.01	0.04	0.03	−0.06	−0.02

Note. RT: response times; con = congruent; incon = incongruent. Response times are measured in milliseconds and standard deviations are in parentheses. Positive priming effects reflect facilitation for targets preceded by high-social, relative to low-social, conditions, such that there are faster RTs and higher accuracy for high-social cues than for low-social cues. Conversely, negative values indicate faster RTs and higher accuracy for low-social cues than for high-social cues, which is indicative of inhibition.

#### 3.2.1. Accuracy

All the data were included for accuracy analyses. Performance was at ceiling, making it difficult to compare conditions. We will therefore not discuss accuracy data in any more detail.

#### 3.2.2. Reaction times

Before going into the main hypothesis, we first investigated whether there was a speed–accuracy trade-off. There was a significant negative correlation between RTs and accuracy (*r* = −0.49, *p* < 0.001), indicating participants who responded more quickly were also the most accurate, thus RTs could be used for analyses. We observed that the model for the second experiment did not converge until the item slope was removed, leaving only an intercept, and only the cue type and task as factors by themselves were included as individual factors in the random structure for the participant. The output of this model is shown in table 7.

**Table 7.** The minimal model output for RTs for Experiment *2*.

fixed effects	estimate	std. error	2.5% CI	97.5% UCI	*t*-values
*A:* (intercept)	−0.6133	0.0431	−0.6989	−0.5276	−14.23^*^
*manipulation*					
intention	−0.0024	0.0273	−0.0561	0.0514	−0.09
task	−0.0052	0.0490	−0.1022	0.0919	−0.11
cue type	−0.0497	0.0277	−0.1043	0.0049	−1.79.
spatial congruency	−0.0205	0.0257	−0.0708	0.0299	−0.80
*interactions*					
intention * task	−0.0821	0.0365	−0.1538	−0.0105	−2.25^*^
intention * cue type	−0.0412	0.0406	−0.1220	0.0395	−1.02
task * cue type	−0.0307	0.0366	−0.1026	0.0411	−0.84
intention * spatial congruency	0.0382	0.0364	−0.0330	0.1095	1.05
task * spatial congruency	−0.0891	0.0365	−0.1606	−0.0175	−2.44^*^
cue type * spatial congruency	−0.0830	0.0362	−0.1540	−0.0120	−2.29^*^
intention * task * cue type	−0.0721	0.0514	−0.0286	0.1728	1.40
intention * task * spatial congruency	0.1895	0.0517	0.0880	0.2909	3.66^*^
intention * cue type * spatial congruency	0.1111	0.0513	0.0106	0.2117	2.82^*^
task * cue type * spatial congruency	0.1450	0.0514	0.0443	0.2458	2.17^*^
intention * task * cue type * spatial congruency	−0.1826	0.0728	−0.3253	−0.0399	−2.51^*^

Note. ^*^ *p* < 0.05. UCI: upper confidence interval.

Although the reduced model was not significantly different from the full model (full model BIC: 4221.4; reduced model = BIC: 2184, *p* = 0.001, ΔBIC: 2037.37, approx. BF > 10 000), the reduced model produced an approximate Bayes factor above 10 000 and a higher ΔBIC value than the full model, suggesting that the removal of these variables improved the model fit and that the reduced model is more likely to occur at least more than 10 000 times than the full model. The final model is therefore based on the reduced model.

#### 3.2.3. Confirmatory analyses

**Figure 3.**
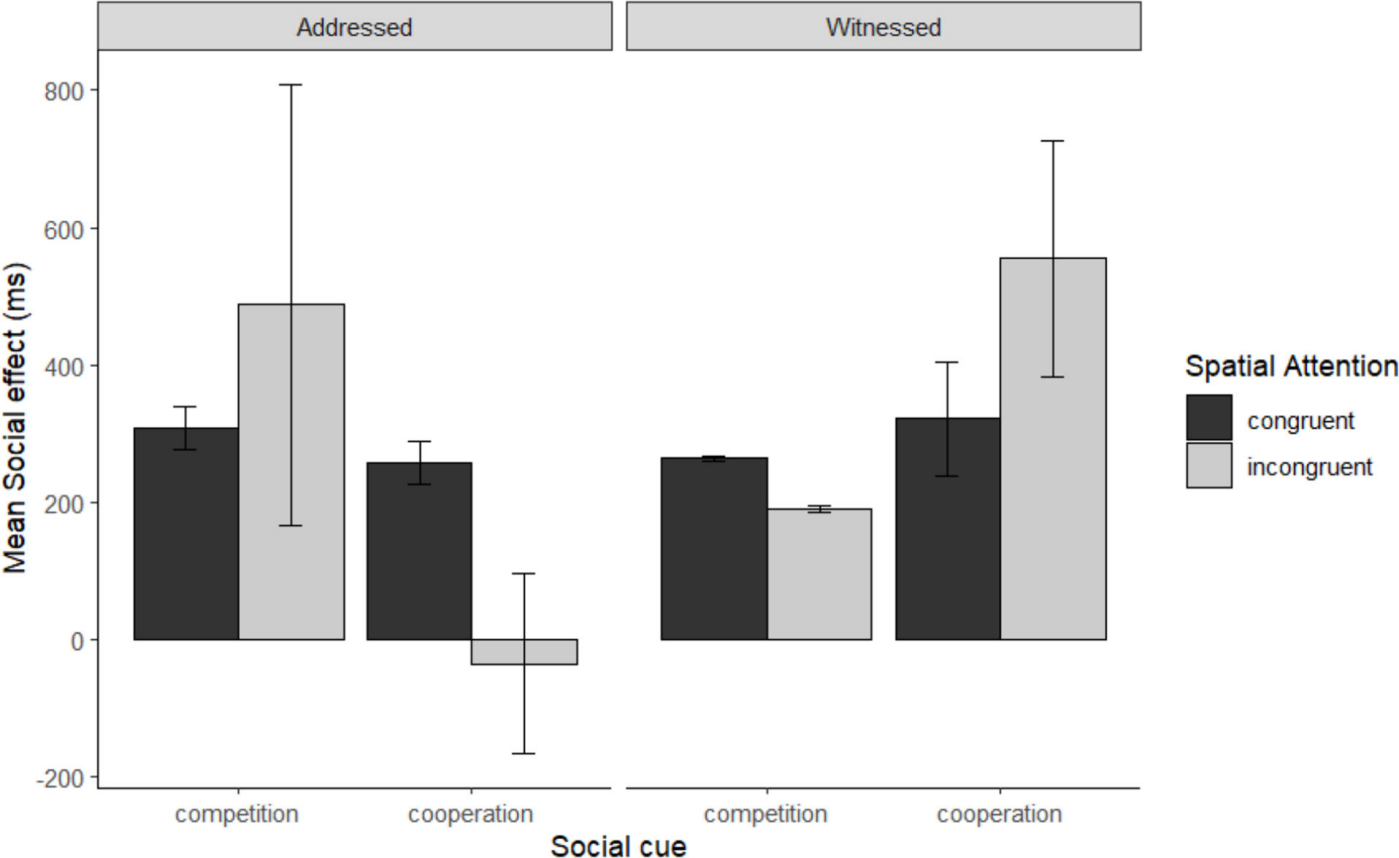
Reaction time (RT) social effects (in ms) in social cues, spatial attention and separated by communicative modality. The Social effect is high-social condition subtracted from the low-social condition. Error bars represents 95% confidence interval for each conditions. Note. Positive priming effects reflect facilitation for targets preceded by high-social, relative to low-social, conditions, such that there are faster RTs and higher accuracy for high-social cues than for low-social cues. Conversely, negative values indicate faster RTs and higher accuracy for low-social cues than for high-social cues, which is indicative of inhibition.

To compare whether there was a difference between cooperative and competitive intentions in addressed and witnessed conditions moderated by spatial congruence, we investigated the four-way interaction of spatial congruence, cue type, task and intention. In the reduced model (table 7), we observed that this interaction was significant (*b* = −0.18, standard error = 0.081, *t* = 2.18, *p* = 0.03) (see figure 3).

To unpack this interaction, we split the reduced model into two sub-models: addressed and witnessed models. We will first focus on the addressed sub-model. Here we removed task from the equation and the same procedures for the analyses and random structures from the reduced model were applied to the sub-model. In the addressed interaction, there was interaction of cue type, intention and spatial congruence (*b* = 0.11, *t* = 2, *p* = 0.02). To unpack this interaction, we split the three-way interaction further into two sub-models: cooperative and competitive. Interaction was removed from the equation and the same procedures for the analyses and random structure from the full model were applied to the sub-models. In the competitive condition, there was no interaction between cue type and spatial congruence (*b* = 0.03, *t* = 0.82, *p* = 0.41), no effect of spatial congruence (*b* = 0.02, *t* = 0.80, *p* = 0.42). There was only an effect of cue type (*b* = −0.09, *t* = −3.32, *p* = 0.002) such that high-social cues were recognized more quickly than low-social cues.

In the cooperative sub-model, we observed an interaction of cue type and spatial congruence (*b* = −0.08, *t* = −2.35, *p* = 0.02). To unpack the interaction further, we therefore split the cooperative sub-model into two further sub-models: spatially incongruent and spatially congruent trials. There was an effect of cue type in the spatially congruent trials (*b* = −0.13, *t* = 3.52, *p* = 0.005). However, there was no effect of cue type in spatially incongruent trials (*b* = 0.05, *t* = 1.56, *p* = 0.13). In the addressed condition, high-social cues were responded to more quickly than low-social cues in spatially congruent cooperative trials only.

In the witnessed sub-model, we did not observe a three-way interaction of intention, cue type and spatial congruence (*b* = −0.07, *t* = −1.34, *p* = 0.18) nor a cue type and spatial congruence interaction (*b* = 0.06, *t* = 1.68, *p* = 0.09), nor intention and cue type (*b* = 0.03, *t* = 0.75, *p* = 0.46). However, we observed an interaction between intention and spatial congruence (*b* = 0.23, *t* = 5.92, *p* < 0.001). To unpack this interaction, we split the three-way interaction further into two sub-models: cooperative and competitive. Intention was removed from the equation and the same procedures for the analyses and random structure from the full model were applied to the sub-models. There was an effect of spatial congruence in the cooperative condition (*b* = −0.11, *t* = −3.96, *p* < 0.001) and the competitive condition (*b* = 0.12, *t* = 4.45, *p* < 0.001). In the witnessed condition, participants responded more quickly to spatially congruent than spatially incongruent trials in the cooperative condition, while participants responded more slowly to spatially congruent than spatially incongruent trials in the competitive condition.

#### 3.2.4. Exploratory analyses

We also conducted exploratory correlational analyses between the r-CIQ scores and competitive effects from each OCT. Competitive effects were calculated by subtracting competitive mean RTs from cooperative mean RTs for each OCT. A significant moderate negative correlation was found between the addressed competitive effect and r-CIQ scores (*r* = −0.28, *p* = 0.05) meaning those scoring low in self-reported competitiveness were more likely to respond slower to competitive stimuli. However, there was no significant correlation between witnessed competitive effect and r-CIQ scores (*r* = 0.02, *p* = 0.89).

We also conducted exploratory correlational analyses between the individual difference measures and competitive effects from each OCT for spatially congruent and incongruent conditions separately. Competitive effects were calculated by subtracting competitive mean reaction times from cooperative mean reaction times for each OCT and spatial congruence. We observed that there was no significant correlation between competitiveness effect in the spatially congruent a-OCT condition and r-CIQ scores (*r* = 0.18, *p* = 0.23), CATI (*r* = −0.03, *p* = 0.82), BPVS (*r* = 0.17, *p* = 0.25) and Raven (*r* = 0.17, *p* = 0.25). In addition, there was no significant correlation between competitiveness effect in the spatially incongruent a-OCT condition and r-CIQ scores (*r* = 0.03, *p* = 0.83), CATI (*r* = −0.14, *p* = 0.33), BPVS (*r* = −0.05, *p* = 0.73) and Raven (*r* = 0.19, *p* = 0.20) that were noted.

In the spatially congruent witnessed condition, we found that there was a significant relationship between competitiveness effect and CATI (*r* = 0.29, *p* = 0.05), where the higher the score for CATI, the larger the competitiveness effect. In addition, we observed a significant relationship between BPVS (*r* = −0.30, *p* = 0.04) and competitiveness effect, meaning that the higher correlation between receptive vocabulary the lower the competitiveness effect. However, there was no significant correlation between competitiveness effect and r-CIQ scores (*r* = 0.06, *p* = 0.70), and Raven (*r* = −0.09, *p* = 0.93). In addition, there was no significant correlation between competitiveness effect in the spatially incongruent w=OCT condition and r-CIQ scores (*r* = 0.16, *p* = 0.27), CATI (*r* = −0.14, *p* = 0.34), BPVS (*r* = 1.55, *p* = 0.13) and Raven (*r* = 0.04, *p* = 0.81) that were evident.

## 3.3. Discussion

Building on Experiment 1, we investigated how cooperative and competitive interactions, in both addressed and witnessed conditions, were modulated by spatial attention. In the addressed condition, participants responded more quickly to high- than low-social cues in the cooperative condition, particularly when trials were spatially congruent. For competitive cues, participants responded faster to high- than low-social pointing cues. In the witnessed condition, we observed a spatial congruency effect in the cooperative setting, with quicker responses to spatially congruent than spatially incongruent trials. Conversely, in the competitive witnessed setting, responses were faster for spatially incongruent trials.

The distinct patterns across conditions suggest differing drivers for the observed effects. In addressed interactions, the social effect for pointing appears to be primarily driven by its communicative social cues. The spatial congruency effect observed in addressed interactions points to a more complex cognitive integration of spatial information within addressed social interaction. By contrast, findings from the witnessed condition indicate that effects are largely reflective of spatial attention, rather than social attention, except in competitive interactions where the spatial incongruency effect emerged. As a result, it is unclear why this behaviour may occur in addressed, not witnessed, interactions. One explanation could be a potential motor-based congruency effect [60], which suggests that observed actions or cues might automatically activate corresponding motor representations, thereby facilitating or interfering with responses, our experimental set-up minimizes this possibility. Participants used a mouse with their dominant hand, selecting ‘1’ or ‘2’ as responses; these were not directly tied to the agent’s pointing hand or specific left/right spatial locations for cooperative/competitive outcomes, as both outcomes could appear on either side of the screen. Instead, our ‘spatial congruency’ referred to the alignment between the target object’s location and the agent’s pointing direction. This complex interaction, rather than a simple effector-to-effector match, suggests a more nuanced integration of spatial information within direct social interaction. This further supports the notion that while pointing’s impact in addressed interactions stems from both social and spatial cues, any effects in witnessed interactions are more likely to reflect the processing of spatial attention.

## 4. General Discussion

The present investigation addressed whether competition and cooperation moderateJA in addressed and witnessed non-verbal environments, using an OCT. Consistent with the literature and predictions, in the addressed OCT, we found that competitive cues had a larger impact on pointing JA socially than cooperative cues. Consistent with the predictions, in the w-OCT, there was no difference between cooperative and competitive cues in terms of pointing socially. The lack of interaction in Experiment 1 was probably due to spatial congruence not being considered. This pattern of finding is similar to the gaze-cueing effect (e.g. [35]) from the previous literature and can only be observed when spatial attention is considered. They found that in addressed interaction, highly competitive individuals were cued equally by both cooperative and competitive faces, while individuals who are less competitive took longer to respond to competitive cues. This suggests that variations in trait competitiveness might affect how individuals monitor their social environment during addressed interactions only.

The current findings corroborate the results of previous studies that have shown that joint attention is susceptible to social context, spatial attention and interaction. Not only do the findings extend from gaze-cued effects to pointing but this study extends these results in three novel ways. Firstly, our research reveals that the effect of pointing gesture is contingent on the social dynamics between the cuer and either participant or observer of the interaction. We matched the range of cuers or observers on age, gender and ethnicity. Previous studies had shown that age, race, sex and social status (e.g. [61–63]) affected JA, as a result we controlled these factors with the actors. Here, we observed that social intentions (e.g. cooperating or competing with the participant or actor) moderated how pointing gestures are interpreted. However, this is limited only to addressed interaction. Second, beyond addressed interactions, we also explored witnessed interactions. Both types of interactions are common in human life (e.g. [24]). Addressed interactions are more salient, as our social reputation and wellbeing can be affected, while witnessed interactions allow us to observe others’ behaviours and learn from their social strategies, especially regarding their intentions to maximize our chances when encountering the individual.

Third, similar to the gaze-cueing effects, spatial attention plays a crucial role in interpreting pointing gestures. We tend to automatically direct our attention towards the direction of others’ non-verbal cues such as pointing, as it often signals important information or potential opportunities. Our results indicated that this tendency is influenced by the social context, regardless of the individual’s gender or race, as the actors were matched on race and gender. Previous research may have overlooked the significance of spatial attention in joint attention, particularly when considering pointing gestures (e.g. [10,32,64,65]). Our study highlights that spatial attention is more influential in witnessed interactions than in addressed interactions, while spatial attention and social intentions are relevant in addressed interactions. These factors suggest that observers are more attuned to pointing gestures. Spatial attention may matter for pointing gestures, as they have a distinct visual outline that has a stronger impact on JA relative to eye gaze and head movements [57,58]. Index-finger pointing is more spatially precise and intentional, making it a more effective tool for conveying spatial information. Index-finger pointing is a cross-cultural gesture that is reinforced daily and unlike eye movements, which can be unintentional, pointing gestures require conscious effort and are often used strategically (e.g. [66]). This intentional nature of pointing makes it more salient to observers. As a result, spatial attention is more likely to matter for JA and needs to be considered for future research. The interplay between these factors investigated is essential for understanding how joint attention through pointing emerges.

More importantly, unlike previous studies that have used the OCT in children, infants and non-human animals (e.g. [9,10,32]), here we extended the use of OCT to adults as a simple measure of JA. Accuracy was not an appropriate metric to assess adults’ ability to select the correct container. Instead, we measured RT to capture subtle nuances in decision-making related to JA, which may not be detectable through accuracy measures alone. We used pointing gestures, which are likely to require more cognitive processing due to their directional nature and clear communicative intent. Humans have a strong tendency to orient their attention towards the direction of a pointing gesture, as it often signals important information relevant to their goals. Additionally, as adults, we have learnt the conventional meaning of pointing gestures, which are typically cooperative but can be exploited by deceptive individuals (e.g. [57,67]). This allows us to investigate joint attention in both cooperative and competitive contexts and can be used as an additional paradigm on JA for future paradigms, especially in neurodivergent groups (e.g., autism [11]; dyspraxia [68).

The relationship between competitiveness and the impact of competitive cues in witnessed and addressed interactions is unexpected. We found a negative correlation between competitiveness effect from OCT and the effect of competition, but only in addressed interactions for Experiment 1. In addressed interactions, the competitive context may require observers to closely monitor others’ actions to gauge their progress towards the same goal but with competing interests [69]. Following a competitor’s pointing gesture can be advantageous, as it allows observers to anticipate their intentions and plan their own actions accordingly. This suggests that individuals may be more attuned to those who are socially significant (e.g. [70]), as they may either aid or hinder the observer’s goals. Supporting evidence has demonstrated that compared with cooperative individuals, non-cooperative individuals attract more automatic attention (e.g. [71]), faces of cheaters are often remembered more vividly than cooperative individuals, including partners (e.g. [71–73]). This might be because we unconsciously connect our ideas about trustworthiness and personality to people who cooperate and compete with us. The present findings and previous evidence support the proposal that pointing-induced attention is enhanced in low-trust situations, similar to what was observed in gaze-cueing effect [35], but only in addressed interactions and when spatial congruence is manipulated.

However, it remains unclear why the correlation between competitiveness and the effect of competitive cues is not observed in witnessed interactions. One possible explanation is that participants must monitor the goals of two individuals with whom they have a low relational value and from whom they do not directly benefit. In this situation, observers may prioritize their own goals over supporting the victim of cheating, especially when the victim’s identity is not identifiable [74]. Therefore, processing pointing cues in addressed interactions may be more cognitively demanding for the recipient compared with observers in witnessed interactions. This increased cognitive load may stem from the additional concern of being directly deceived and facing potential social consequences, which is not a concern in witnessed interactions. This pattern of results was observed regardless of spatial congruence.

### 4.1. Limitations

While no interaction was shown in Experiment 1 but observed in Experiment 2, our understanding of the influence of competition and cooperation on JA in an addressed and witnessed interaction remains limited. Results obtained in an artificial environment might not necessarily reflect the complexities of real-world decision making. In the Global South, JA is primarily driven by witnessed interactions more than addressed interactions. As a result, it is unclear whether these spatial factors observed would contribute to JA in both addressed and witnessed interactions in the Global South. Future studies can replicate our pattern of findings and assess whether the findings are specific to that of the Global North or are more universal and applicable to the Global South.

One potential limitation of the current study lies in the comparison between the ‘witnessing’ and ‘addressed’ conditions. Specifically, the number of agents present in the scene was not matched, with only one agent in the ‘addressed’ condition compared with two agents in the ‘witnessed’ condition. This disparity in the number of agents could be considered a confound in the experimental stimuli. We acknowledge that this introduces an additional layer of complexity in the ‘witnessed’ condition due to the presence of an extra agent and the dynamic of agent–agent interaction, which is absent when an agent is directly addressing the participant. This inherent difference in agent count could be considered a confound in the stimuli, as it may contribute to the observed effects beyond the intended manipulation of witnessing an event versus being directly addressed. However, our methodological approach directly follows previous literature and established paradigms in this area of research. These prior studies (e.g. [10,32,65]), which our work builds upon, have similarly used stimuli where the ‘witnessed’ condition inherently involves an additional agent (e.g. an interaction between two agents), thereby introducing an ‘extra agent–agent directly with you’ scenario not present in the ‘addressed’ condition. While acknowledging this as a potential confound, we opted to maintain consistency with these validated paradigms to allow for direct comparison and integration of our findings within the existing body of literature. Future research could explore alternative stimulus designs that more tightly control for the number of agents across conditions, while still capturing the essence of witnessed versus addressed social interactions.

Even though we observed patterns similar to previous studies in OCT (see review by Clark *et al*. [37]), the OCT was conducted online. The findings of the present study may require further generalizability in more ecological settings. The OCT is typically administered as an in-person task, which may be richer in terms of attention and experience. While acknowledging the limitations of our study’s online environment [75], we believe the observed correlation between joint attention and competitiveness holds relevance in real-world settings. Participants with higher competitiveness were more likely to show smaller competitive joint attention effects. Here, trait competitiveness is known to contribute in goal pursuit and behaviour in achievement situations, such as competitive context [76]. However, we also recognize that the specific expression of this relationship might differ in real-world contexts due to the influence of additional factors like social norms, organizational culture and interpersonal dynamics. As a result, future-targeted studies should address this issue by comparing in-person and online performance to assess whether JA differs between modalities, perhaps using a creative destruction approach (i.e. pre-specifying alternative results by competing hypotheses on a complex set of experimental findings; [77–79]). More work is needed to extend to real-world in-person interactions.

## 5. Conclusion

We sought to test whether competition and cooperation modulates JA in an addressed and witnessed environment. Contrary to previous findings, In Experiment 1, we found no interaction between intention, task and cue type, suggesting that the social components and pointing have additive effects. However, when spatial congruence was included as an additional factor due to pointing’s directional nature, interactions among intention, task and cue type emerged. We observed that JA in addressed environments is more driven by social and spatial influences, while JA in witnessed environments is primarily driven by spatial influences. Overall, our study indicates that the OCT is a suitable task to assess JA in adults but also that JA via pointing is driven by an interaction of social and spatial attention. Future research should aim to involve larger and more diverse samples to increase the generalizability of the results.

## Data Availability

The data and analysis are located here: https://osf.io/87cx5/. Supplementary material is available online [80].
